# Analysis and validation of carbohydrate three-dimensional structures

**DOI:** 10.1107/S0907444909001905

**Published:** 2009-01-20

**Authors:** Thomas Lütteke

**Affiliations:** a Bijvoet Centre for Biomolecular Research, BOC2, Utrecht University, Padualaan 8, 3584 CH Utrecht, The Netherlands; b Centre for Molecular and Biomolecular Informatics, Nijmegen Centre for Molecular Life Sciences, Radboud University Nijmegen, PO Box 9101, 6500 HB Nijmegen, The Netherlands

**Keywords:** validation, glycosylation, glycan conformation, three-dimensional structure quality

## Abstract

Knowledge of the three-dimensional structures of the carbo­hydrate molecules is indispensable for a full understanding of the molecular processes in which carbohydrates are involved, such as protein glycosylation or protein–carbohydrate interactions. The Protein Data Bank (PDB) is a valuable resource for three-dimensional structural information on glycoproteins and protein–carbohydrate complexes. Unfortunately, many carbohydrate moieties in the PDB contain inconsistencies or errors. This article gives an overview of the information that can be obtained from individual PDB entries and from statistical analyses of sets of three-dimensional structures, of typical problems that arise during the analysis of carbohydrate three-dimensional structures and of the validation tools that are currently available to scientists to evaluate the quality of these structures.

## Abstract 2

The article summarizes the information that is gained from and the errors that are found in carbohydrate structures in the Protein Data Bank. Validation tools that can locate these errors are described.

## 1. Introduction

### 1.1. Protein glycosylation

Carbohydrates, often referred to as glycans, play an im­portant role in many biological and biochemical processes, ranging from protein folding to a variety of recognition events, many of which are of immunological importance (Varki *et al.*, 1999 ▶; Helenius & Aebi, 2001 ▶; Ohtsubo & Marth, 2006 ▶). Of the co-translational and post-translational modifications of proteins, such as phosphorylation, glycosylation or acetyl­ation, glycosylation is probably by far the most common and the most complex (Helenius & Aebi, 2001 ▶; Charlwood *et al.*, 2001 ▶). Glycosylation is classified by the way the carbohydrate chain is linked to the protein. The best understood subclass is *N*-glycosylation, in which the glycans are linked to the N^δ2^ atom of an Asn side chain. A prerequisite for *N*-glycosylation is the sequence motif Asn-Xaa-Ser/Thr (where Xaa can be any amino acid except for Pro), the so-called sequon (Marshall, 1972 ▶). This motif is found in about two-thirds of all proteins (Apweiler *et al.*, 1999 ▶). For *O*-glycosylation, which occurs when a glycan chain is linked to an O atom of a free hydroxyl group (mostly of a Ser or Thr side chain), no such consensus sequence motif is known (Julenius *et al.*, 2005 ▶). Not all of the potential glycosylation sites are actually occupied in nature, but nevertheless more than 50% of all proteins in nature have been estimated to be glycosylated (Apweiler *et al.*, 1999 ▶). Protein glycosylation fulfils a variety of roles. The glycan chains alter the properties of the proteins to which they are attached, making them more soluble (Jones *et al.*, 2005 ▶) and protecting them from proteolysis (Garner *et al.*, 2001 ▶; Indyk *et al.*, 2007 ▶), and also influence protein stability (see §[Sec sec1.2]1.2). Furthermore, they serve as recognition motifs in protein trafficking (Guo *et al.*, 2004 ▶; Shi & Elliott, 2004 ▶; Hart *et al.*, 2007 ▶) or to mark proteins for clearance from circulation (Ashwell & Harford, 1982 ▶; van Rensburg *et al.*, 2004 ▶; Jones *et al.*, 2007 ▶). Hereditary dysfunctions in the glycosylation machinery, called congenital disorders of glycosylation (CDG), lead to severe phenotypic problems (Jaeken & Matthijs, 2001 ▶; Ye & Marth, 2004 ▶; Freeze, 2006 ▶).

Carbohydrates differ from proteins in two important features. The first difference is found in the primary structures. The number of different building blocks available, the monosaccharides, is much larger than the number of different amino acids (Berteau & Stenutz, 2004 ▶) and the monosaccharides can be linked in various ways, with the possibility of forming branched structures (Schachter, 2000 ▶). In a recent analysis of various carbohydrate databases, about three-quarters of all entries contained at least one branching position (Werz *et al.*, 2007 ▶). Therefore, carbohydrate chains are usually displayed as a tree-like two-dimensional graph. In glycobiology, the term ‘structure’ is mainly used to describe such a two-dimensional graph and not, as in crystallography, the three-dimensional structure of a molecule. To avoid confusion, the simple term ‘structure’ is avoided in this article. Instead, ‘primary structure’ and ‘three-dimensional structure’ are used to distinguish between ‘structure’ in the glycobiological sense and ‘structure’ in the crystallographic sense, respectively.

The second major difference between carbohydrates and proteins lies in their biosynthesis. Unlike proteins, the glycans are indirectly encoded in the genome (Varki *et al.*, 1999 ▶). Depending on the tissue, the developmental age and the health/disease state of a cell, different glycosyltransferases, the enzymes that build the glycans in a non-template-driven fashion, are expressed (Kornfeld & Kornfeld, 1985 ▶; Schachter, 2000 ▶; Esko & Selleck, 2002 ▶; Ohtsubo & Marth, 2006 ▶). This results in different primary structures of the glycans and thus allows a ‘fine-tuning’ of proteins (Helenius & Aebi, 2001 ▶; Drescher *et al.*, 2003 ▶).

The glycan chains found on a protein do not only differ between different organisms, tissues or cells, but various different glycans can also be present on one type of protein in one single cell, tissue or organism (Rudd & Dwek, 1997 ▶). The resulting isoforms of the protein are called glycoforms (Parekh *et al.*, 1987 ▶). The GPI-anchored protein CD59, for example, consists of a heterogeneous mixture of more than 120 glycoforms (Rudd *et al.*, 1997 ▶).

### 1.2. Influence of glycosylation on protein folding and conformation

N-linked glycans can affect the protein structure in two capacities. Firstly, *N*-glycosylation occurs co-translationally and plays an important role during the folding process and in the detection of incorrectly folded proteins in the calnexin–calreticulin cycle (reviewed in Parodi, 2000 ▶; Schrag *et al.*, 2003 ▶; Molinari, 2007 ▶). Secondly, the glycan chains have a stabilizing effect on the structure of the mature protein (Wormald *et al.*, 1991 ▶; Live *et al.*, 1996 ▶; van Zuylen *et al.*, 1997 ▶; Imperiali & O’Connor, 1999 ▶; Bosques *et al.*, 2004 ▶). Glycans attached to peptides decrease the conformational mobility of the peptide backbone (Bailey *et al.*, 2000 ▶). The degree of thermal stabil­ization depends on the position of the glycosylation sites, but only weakly on the size of the glycan chains (Shental-Bechor & Levy, 2008 ▶). In some cases, glycosylation can have such an impact on stabilizing the protein conformation that in the absence of the glycan chain, receptors no longer properly interact with their ligands, even though the glycosylation site is located opposite the ligand-binding site (see §[Sec sec2.1]2.1). Contradictory results have been found for the effect of *O*-glycosylation on peptide stability. While *O*-glycosylation can increase the stability of helices in peptides (Palian *et al.*, 2001 ▶), there are also studies that have reported a destabilizing effect of *O*-­glycosylation on some peptides (Vijayalekshmi *et al.*, 2003 ▶; Spiriti *et al.*, 2008 ▶).

### 1.3. Protein–carbohydrate interactions

In addition to their impact on glycoproteins, carbohydrates play an important role in a variety of cell–cell and cell–matrix interactions (Lis & Sharon, 1998 ▶). Glycans on cell surfaces are already involved in many important metabolic processes in the early development of an organism, such as fertilization (Rosati *et al.*, 2000 ▶; Diekman, 2003 ▶) and cell differentiation and maturation (Moody *et al.*, 2001 ▶; Haltiwanger & Lowe, 2004 ▶; Lau *et al.*, 2007 ▶). Later on, they participate, for example, in processes such as apoptosis (Martinez *et al.*, 2004 ▶; Tribulatti *et al.*, 2007 ▶; Suzuki & Abe, 2008 ▶), blood clotting (Tenno *et al.*, 2007 ▶), inflammation (Brinkman-van der Linden *et al.*, 1998 ▶; Sharon & Ofek, 2000 ▶), host–pathogen interactions (Smith & Helenius, 2004 ▶; Lehr *et al.*, 2007 ▶), the immune response (Kogelberg & Feizi, 2001 ▶; Klement *et al.*, 2007 ▶; van Kooyk & Rabinovich, 2008 ▶) and diseases such as arthritis, Alzheimer’s disease and cancer (Hakomori, 2002 ▶; Lahm *et al.*, 2004 ▶; Kobata & Amano, 2005 ▶; Mendelsohn *et al.*, 2007 ▶; Nakahara & Raz, 2008 ▶). Their implications for the immune response make them interesting targets for vaccine development (Vliegenthart, 2006 ▶). All these processes require a precise recognition of the carbohydrate by the carbohydrate-binding proteins. The same applies to glycosyltransferases and glycosidases, the enzymes that build or degrade the carbohydrate chains, respectively. These enzymes must recognize their substrates precisely. The three-dimensional structures of carbohydrate–protein com­plexes can help us to understand the mechanisms of the distinction even between very similar carbohydrate residues, which often only differ in the stereochemistry of one or two C atoms.

## 2. Analysis of carbohydrate and glycoprotein three-dimensional structures

Knowledge of the three-dimensional structures of glyco­proteins or protein–carbohydrate complexes is often indispensable for a full understanding of the molecular processes that carbohydrates are involved in. Insights into the key interactions between lectins or carbohydrate-processing enzymes and their ligands are also required for the targeted development of drugs that inhibit these interactions (Lovering *et al.*, 2007 ▶). Therefore, X-ray crystallography (*e.g.* Delbaere, 1974 ▶; Jain *et al.*, 1996 ▶; Mølgaard & Larsen, 2002 ▶; Stevens *et al.*, 2004 ▶; Fry *et al.*, 2005 ▶; Smith *et al.*, 2006 ▶; Vulliez-Le Normand *et al.*, 2008 ▶) and NMR (*e.g.* Brisson & Carver, 1983 ▶; Cumming *et al.*, 1987 ▶; Sabesan *et al.*, 1991 ▶; Koles *et al.*, 2004 ▶; Petersen *et al.*, 2008 ▶), the latter often in combination with MD simulations (*e.g.* Höög *et al.*, 2001 ▶; Lommerse *et al.*, 2002 ▶; Eklund *et al.*, 2005 ▶; Siebert *et al.*, 2005 ▶), have been used to resolve the three-dimensional structures of carbohydrates, glycoproteins and protein–carbohydrate complexes. X-ray crystallography can also be combined with computational chemistry (Ali *et al.*, 2008 ▶) or NMR (Viegas *et al.*, 2008 ▶). Uncomplexed carbo­hydrate three-dimensional structures are mainly submitted to the Cambridge Structural Database (CSD; Allen, 2002 ▶), while the three-dimensional structures of glycoproteins and protein–carbohydrate complexes can be found in the Protein Data Bank (PDB; Berman *et al.*, 2000 ▶). The following sections will illustrate a few results that were obtained from individual structures and give an overview of attempts to statistically analyse data retrieved from sets of PDB entries.

### 2.1. Information gained from individual structures

The functions of individual glycosylation sites are often poorly understood. Three-dimensional structures can help to obtain insights into these functions. For example, the three-dimensional structure of the intercellular adhesion molecule ICAM-2 reveals that some of its *N*-glycans are arranged in a tripod-like shape and thus are likely to be used to orient the receptor on a cell surface (Casasnovas *et al.*, 1997 ▶). Although the integrin-binding domain of ICAMs is glycan-free (Shi­maoka *et al.*, 2003 ▶), deletion of the glycosylation site at Asn23 largely decreased the binding of the leukocyte integrin LFA-1 (Jiménez *et al.*, 2005 ▶). The three-dimensional structure of this molecule shows that the proximal β-d-Glc*p*NAc of the glycan chain linked to Asn23 stacks on the aromatic ring of Trp51. This interaction contributes to the protein conformation in a way that is essential for integrin binding by ICAM-2, even though the glycan-Trp motif is located on the opposite side of the interacting surface (Jiménez *et al.*, 2005 ▶). A similar effect is observed for human CD2, which is a cell-surface protein that is present on T lymphocytes and natural killer cells. Human CD2 no longer binds to its counter-receptor CD58 after the removal of a glycan chain opposite the binding site (Recny *et al.*, 1992 ▶). In this case, the glycan chain covers an area of five surface-exposed Lys residues. Without the shielding carbohydrate, this accumulation of negative charges has a destabil­izing effect on the protein (Wyss *et al.*, 1995 ▶).

The involvement of carbohydrates in many immunological and pathogenic processes makes them a promising target for drug design, which requires knowledge of the three-dimensional structures of the molecules involved (von Itzstein, 2008 ▶). For example, UDP-galactopyranose mutase (UGM) is a key enzyme in the biosynthesis of d-galactofuranose (d-Gal*f*), a monosaccharide that forms part of the cell wall of tuber­culosis-causing mycobacteria and that is essential for their survival and infectivity (Duncan, 2004 ▶). d-­Gal*f* does not occur in mammals (de Lederkremer & Colli, 1995 ▶) and therefore the enzymes involved in its biosynthesis are promising candidates for antimycobacterial drugs (Yuan *et al.*, 2008 ▶). The three-dimensional structures of UGM reveal a mobile loop (Sanders *et al.*, 2001 ▶; Beis *et al.*, 2005 ▶), which acts as an active-site lid during catalysis (Yuan *et al.*, 2008 ▶). This insight opens two directions for inhibitor design: the design of molecules that prevent closure of the loop or of molecules that keep the loop closed (von Itzstein, 2008 ▶).

In some cases, the three-dimensional structural data can reveal novel and unexpected features of proteins. An example is the crystal structure of langerin, a cell-surface receptor with a C-type lectin domain (Chatwell *et al.*, 2008 ▶). A characteristic feature of C-type lectins is a calcium-dependent carbohydrate-recognition domain (Kogelberg & Feizi, 2001 ▶). This three-dimensional structure disclosed a novel calcium-independent carbohydrate-recognition domain in addition to the usual calcium-dependent domain (Chatwell *et al.*, 2008 ▶).

In contrast to information about individual structures, knowledge of general properties of carbohydrates, such as preferred conformations, can only be gained from studies of sets of three-dimensional structures, which will be the subject of the following section.

### 2.2. Statistical analyses of sets of three-dimensional structures

Oligosaccharides are much more flexible than proteins or nucleic acids (Woods, 1998 ▶; Frank *et al.*, 2002 ▶). Single three-dimensional structures are therefore only a static snapshot of one of the various conformations, which might not correspond to the average solution conformation. However, sufficiently large samples of such static three-dimensional structures can yield information on the conformations that are possible for an oligosaccharide and the flexibility of the linkages, provided that no systematic changes in the linkage conformations are imposed by packing forces in the available crystals (Petrescu *et al.*, 1999 ▶).

Access to the complete data set of three-dimensional structures in the CSD is restricted to institutes paying a license fee, whereas the data in the PDB are freely accessible. Therefore, the analyses presented here are all based on data from the PDB. There have been several attempts to gain information on the properties of carbohydrates from statistical examinations of PDB entries, with a main focus on *N*-glycans (Imberty & Pérez, 1995 ▶; Petrescu *et al.*, 1999 ▶, 2004 ▶; Wormald *et al.*, 2002 ▶). As the monosaccharide rings are rather rigid, the conformation of a glycan chain can be classified by the torsion angles of the rotatable bonds, mainly the ϕ and ψ torsions of the glycosidic linkages (Wormald *et al.*, 2002 ▶). For (1–6)-linked residues, there is an additional rotatable bond classified by the ω torsion (Cumming & Carver, 1987 ▶). In the literature, several different definitions of these torsions can be found. Therefore, one always needs to check which definition has been used in a single study. Table 1 ▶ lists three frequently used definitions. The first makes use of H atoms. These are mainly seen in three-dimensional structures that have been resolved by NMR; therefore, this definition is sometimes referred to as ‘NMR type’. H atoms typically cannot be resolved in X-ray structures. To measure torsions in such a three-dimensional structure, the ring O atom is used instead of the H_1_ atom in the definition of the ϕ angle, while for the ψ angle either the ring C atom preceding (‘C − 1 crystallographic definition’) or that following (‘C + 1 crystallographic definition’) the ring C atom at the linkage position is used. The values observed for the different definitions can be converted into each other by adding or subtracting 120°, depending on the stereochemistry of the ring C atoms involved. A web tool is available to perform such conversions (http://www.dkfz.de/spec/ppc/). In this article, the C + 1 crystallographic definition is used (see Table 1 ▶).

**Figure 1:**
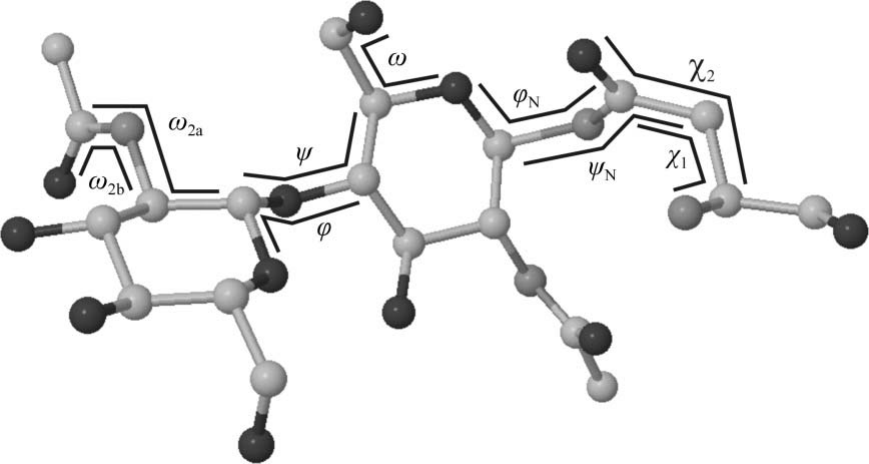
Definition of glycosidic torsion angles used in this article.

**Figure 2:**
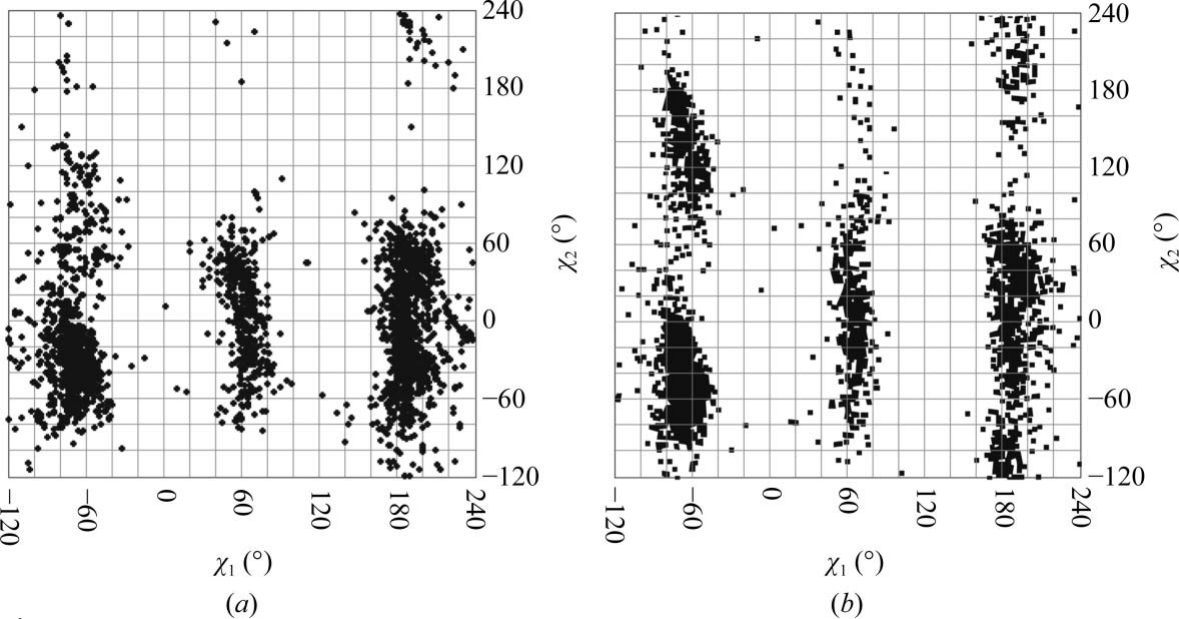
Asn side-chain torsions of occupied *N*-glycosylation sites (*a*) and all Asn side chains (*b*). Glycosylation limits the conformational range of the χ_2_ angle and changes the relative frequencies of the three staggered conformations of the χ_1_ angle. The plot containing the occupied sites was created with *glyTorsion* (http://www.glycosciences.de/tools/glytorsion/; Lütteke *et al.*, 2005 ▶); that containing all Asn side chains was taken from the Conformation Angles Database (http://144.16.71.148/cadb/; Sheik *et al.*, 2003 ▶).

The first investigation of carbohydrate structures from the PDB was performed by Imberty & Pérez (1995 ▶). They analysed the torsion angles of 44 *N*-glycan chains taken from 29 PDB entries, focusing on the linkages between Asn and the proximal β-d-Glc*p*NAc residue, the Asn side-chain torsions and the ω_2_ and ω_6_ torsions (see Fig. 1 ▶) of β-d-Glc*p*NAc and the backbone conformations of the glycoproteins. Almost a decade later, Petrescu *et al.* (2004 ▶) performed a similar analysis using 1683 *N*-glycosylation sites. Both studies observed torsion angles of the β-d-Glc*p*NAc-(1–*N*)-Asn linkage of about −90° for ϕ_N_ and about 180° for ψ_N_, with ϕ_N_ occupying a broader range of conformations than ψ_N_ (see Fig. 7*a*). These results correspond well to the values measured from small-molecule crystal structures of analogues of this linkage (Lakshmanan *et al.*, 2003 ▶). Comparison of the Asn side-chain torsions of occupied and unoccupied *N*-glycosyl­ation sites only revealed noticeable differences in the latter study. Both occupied and unoccupied Asn side chains exhibit χ_1_ torsion angles of −60°, 60° or 180°, corresponding to the *g* ^−^, *g* ^+^ and *t* conformers (Janin & Wodak, 1978 ▶), respectively. The χ_2_ torsion angle (N^δ^—C^γ^—C^β^—C^α^) does not display these threefold staggered conformations because the Asn C^γ^ atom is not a tetrahedral C atom. Instead, it shows a wide distribution centred at about 180° (or 0° when defined as C^α^—C^β^—C^γ^—O as in the study by Imberty and Pérez). This distribution is much smaller for glycosylated Asn than for nonglycosylated Asn residues (see Fig. 2 ▶). Furthermore, the relative populations of the three conformers change upon glycosylation. In an unoccupied Asn side chain the *g* ^−^ conformer is preferred over the *t* conformer, whereas in occupied Asn the *t* conformer is found more frequently than the *g* ^−^ conformer. The *g* ^+^ conformer is the rarest in both glycosylated and non­glycosylated Asn residues (Petrescu *et al.*, 2004 ▶). Using the small data set that was available in 1995 these differences could not been seen, so Imberty and Pérez assumed at that time that *N*-­glycosylation does not have a significant effect on Asn side-chain conformation. These examples show that even rather small data sets can yield information on preferred conformations of glycosidic linkages, but that some specific properties may only be seen in larger data sets.

**Figure 3:**
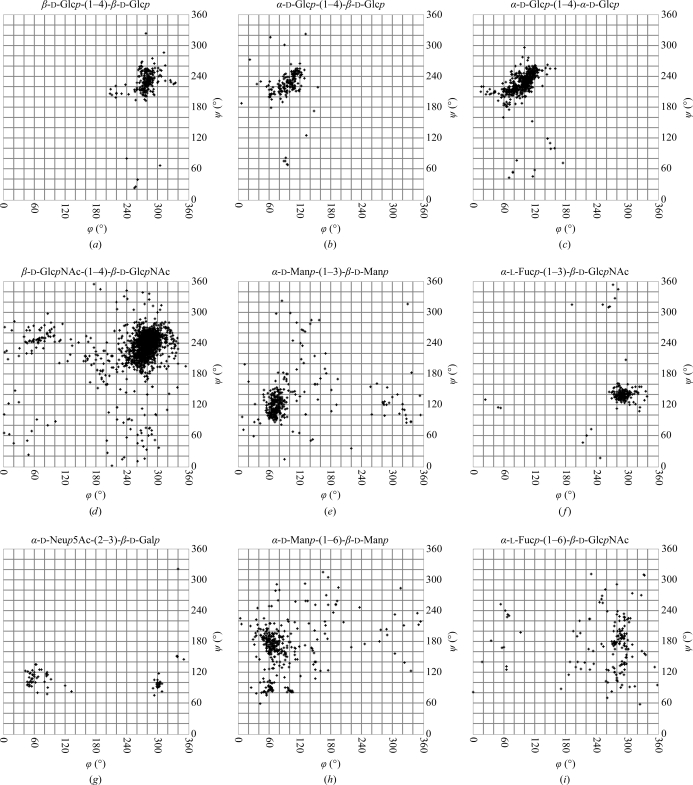
Comparison of glycosidic torsions as present in the PDB. It becomes obvious that the residues involved in the linkage as well as the linkage position can influence the preferred conformation. The plots were generated with *glyTorsion* (see the legend to Fig. 2 ▶). Number of torsions per plot: (*a*) 247, (*b*) 454, (*c*) 1356, (*d*) 2755, (*e*) 162, (*f*) 211, (*g*) 76, (*h*) 362, (*i*) 162.

**Figure 4:**
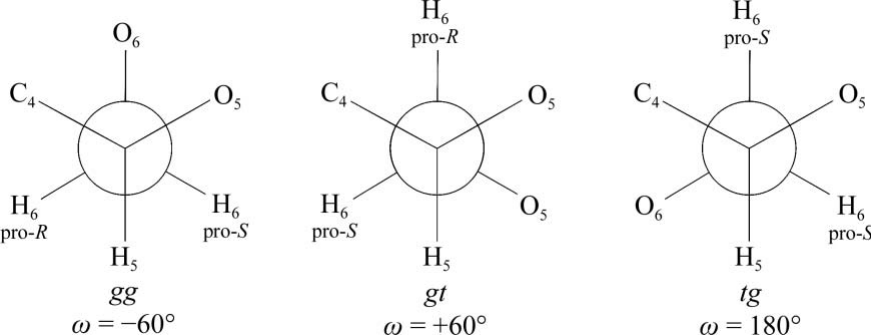
Definition of ω_6_ conformations. The ω_6_ torsion (O_6_—C_6_—C_5_—O_5_) mainly occurs in one of the three staggered conformations, which are often referred to as the *gauche*–*gauche* (*gg*), *gauche*–*trans* (*gt*) and *trans*–*gauche* (*gt*) rotamers (adapted from Wyss *et al.*, 1995 ▶).

**Figure 5:**
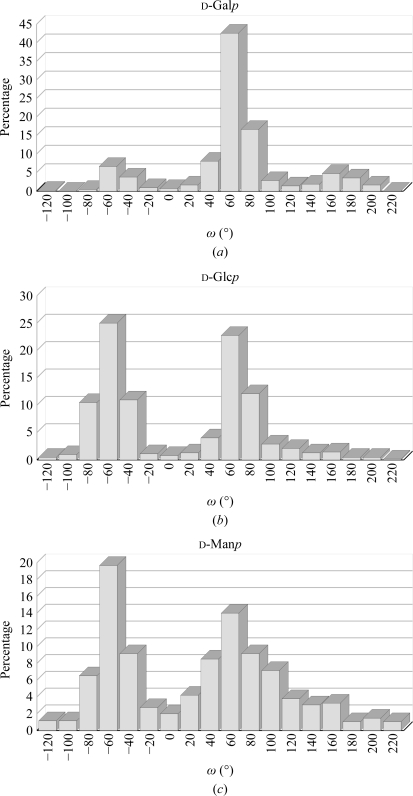
Conformational analysis of ω_6_ torsions as a function of the monosaccharide type. An axial hydroxyl group linked to the C_4_ atom promotes the *gt* conformation (*a*), while in residues with an equatorial hydroxyl group in this position both the *gg* and the *gt* conformations are populated (*b*, *c*). The diagrams were created with *glyTorsion* (see the legend to Fig. 2 ▶).

Analysis of the torsion angles of various kinds of glycosidic linkages revealed that both the preferred torsions and the degree of conformational dispersion depend on the linkage position and the participating monosaccharide residues (Petrescu *et al.*, 1999 ▶; Wormald *et al.*, 2002 ▶). Fig. 3 ▶ shows the torsions of various linkages as present in the current version of the PDB. In this figure, as in Figs. 2, 5 and 7, only structures with a resolution of 3.0 Å or better were analysed. Furthermore, residues with mismatches between the PDB residue name and the residue type present in the three-dimensional structure (see §[Sec sec3]3) were omitted. Changing the stereochemistry of the anomeric centre (the atom to which the ring O atom is linked during ring closure; usually the C1 atom) involved in the linkage from α to β results in a shift of the ϕ angle of about 180° (Figs. 3 ▶ *a* and 3 ▶ *b*). In contrast, the anomer of the proximal residue does not have any significant influence on the conformation of a (1–4)-linkage (Figs. 3 ▶ *b* and 3 ▶ *c*). The *N*-acetyl groups of the β-d-Glc*p*NAc-(1–4)-β-d-Glc*p*NAc fragment also do not significantly affect the linkage torsions in comparison with the non-acetylated residues (Figs. 3 ▶ *a* and 3 ▶ *d*). It also becomes obvious from this figure that the various linkages exhibit a different degree of conformational flexibility. While for α-l-Fuc*p*-(1–3)-β-d-Glc*p*NAc linkages rather little dispersion is seen, α-d-Man*p*-(1–3)-β-d-Man*p* linkages cover a broader range of torsion angles (Figs. 3 ▶ *e* and 3 ▶ *f*). For α-­d-Neu*p*5Ac-(2–3)-β-d-Gal*p* linkages, two distinct conformations are clearly visible in the ϕ/ψ plot (Fig. 3 ▶ *g*). Three energy minima are known for this linkage (Siebert *et al.*, 2003 ▶), but only two of them are observed in the PDB. As a result of the additional rotatable bond, most scatter is seen with 1–6 linkages (Fig. 3 ▶ *h* and 3 ▶ *i*). In addition to the residues involved and the linkage type, the degree of flexibility also depends on the degree of branching of a carbohydrate chain, as neighbouring branches often limit the conformational space that is accessible to a linkage (Frank *et al.*, 2007 ▶). Three staggered conformations are possible for the ω_6_ torsion. They are named *gg*, *gt* and *tg* (see Fig. 4 ▶). In monosaccharides with an axial OH group at position 4, such as d-Gal*p*, the *gt* conformation is most frequently observed, while monosaccharides with an equatorial 4-OH group, such as d-Glc*p* or d-Man*p*, prefer the *gg* and *gt* conformations (Petrescu *et al.*, 1999 ▶; Fig. 5 ▶).

The carbohydrate data present in the PDB not only enable the study of the conformations of *N*-glycans but also of non­covalently bound ligands. For instance, a statistical analysis of glycosaminoglycan (GAG) chains in the PDB revealed that binding of the GAG chains to receptor proteins induces a kink in the GAG backbone to provide optimal ionic and van der Waals contacts between the protein and the oligosaccharide (Raman *et al.*, 2003 ▶).

The rapid growth of the PDB and the concomitant growth in carbohydrate three-dimensional structures requires the development of algorithms to automatically detect carbo­hydrate components in PDB entries, as the PDB itself does not provide any methods for a targeted search for carbohydrates. Two such projects have been published to date. The first was the *pdb*2*linucs* software (Lütteke *et al.*, 2004 ▶), which can be accessed through the glycosciences.de web portal (http://www.glycosciences.de; Lütteke *et al.*, 2006 ▶). This software searches the three-dimensional structure file for rings, selects potential carbohydrate rings using a set of criteria (*e.g.* the number of C and O atoms in the ring, nonplanarity and the existence of exocyclic O atoms) and then builds a stereocode string to identify the monosaccharide residue type of these rings (Lütteke *et al.*, 2004 ▶). The detected carbohydrate chains are given in LINUCS notation, a linear and unique description of carbohydrate chains (Bohne-Lang *et al.*, 2001 ▶). The im­plementation of these data into the glycosciences.de database (Lütteke *et al.*, 2006 ▶), the former SweetDB (Loss *et al.*, 2002 ▶), provided the first possibility for glycoscientists to perform a targeted search for carbohydrate chains in PDB entries. The second project that aims to detect carbohydrates in three-dimensional structural data from the PDB is the *getCarbo* software (Nakahara *et al.*, 2008 ▶). This software uses an algorithm similar to that used by *pdb*2*linucs*. The detected carbohydrate chains are stored in the GDB:Structures database (Nakahara *et al.*, 2008 ▶).

About 7% of the three-dimensional structures deposited in the PDB contain carbohydrate residues (Table 2 ▶). The vast majority of the carbohydrate chains that are present in the PDB are *N*-glycans or noncovalently bound ligands. *O*-Glycan chains form a minority (Table 2 ▶). In total, about 3.5% of the proteins in the PDB carry covalently bound glycan chains and thus can be classified as glycoproteins. This stands in marked contrast to the assumption that more than 50% of all proteins are glycosylated (Apweiler *et al.*, 1999 ▶). There are multiple reasons for the relatively low rate of glycosylated proteins among PDB entries. Firstly, glycan chains often hamper crystal growth and thus are often removed by glycosidases beforehand (Imberty & Pérez, 1995 ▶; Chang *et al.*, 2007 ▶). Secondly, the proteins to be used for crystallization are often purified from bacterial expression systems. Most of these do not have glycosylation machinery or have machinery that differs from that of eukaryotic species (Szymanski & Wren, 2005 ▶; Kowarik, Young *et al.*, 2006 ▶; Kowarik, Numao *et al.*, 2006 ▶), so that proteins expressed in bacteria often are not glycosylated, even if the original protein is known to be a glycoprotein *in vivo* (von der Lieth *et al.*, 2006 ▶). Thirdly, as mentioned above, carbohydrates are rather flexible and therefore often do not yield sufficient electron density to be resolved in the three-dimensional structure. The presence of different glycoforms at one *N*-glycosylation site might further contribute to poor electron density. However, this should have only a minor effect, as all *N*-glycan chains share a common core structure. If glycan chains can be resolved, then often only the proximal monosaccharide units which are close to the protein can be seen in the electron-density map, as the degree of mobility of the glycan core is smaller than that of peripheral glycan residues (Lommerse *et al.*, 1995 ▶). This is one of the reasons why almost 80% of the *N*-glycan chains in the PDB consist of only one or two monosaccharide units (Table 3 ▶). Relatively long *N*-glycan chains are mainly found in those cases where contacts between the glycan chain and the protein or crystal contacts immobilize the carbohydrate (Petrescu *et al.*, 1999 ▶). Another reason why often only the first β-d-Glc*p*NAc residue of an *N*-glycan chain is present in the three-dimensional structure file is the fact that sometimes the glycan chains are not completely removed in order to improve crystal growth: proteins are treated with an endoglucanase that cleaves the *N*-­glycan chains after the first monosaccharide (Chang *et al.*, 2007 ▶).

## 3. Erroneous entries

**Figure 6:**
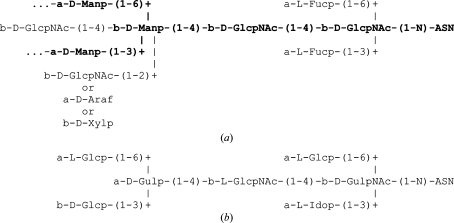
*N*-glycan core structure (with frequent additions) and an example of erroneous PDB data. (*a*) Carbohydrate chains that are linked to an Asn side chain (*N*-glycans) comprise a well defined core structure of two β-d-Glc*p*NAc, one β-d-Man*p* and two α-d-Man*p* residues (‘GlcNAc_2_Man_3_ core’), displayed in bold letters. At positions 3 and/or 6 of the proximal β-d-Glc*p*NAc, α-l-Fuc*p* residues can be added (‘core fucosylation’). Some residues are only present in certain species. For example, the β-d-Xyl*p* and α-d-Ara*f* residues that are linked to position 2 of β-d-Man*p* are found, for example, in insects, molluscs or plants but not in mammals. The core structure can be further extended (mainly by d-Glc*p*NAc, d-Gal*p*, d-Gal*p*NAc, d-Man*p*, d-Neu*p*5Ac, l-Fuc*p* or d-Glc*p*) at the α-d-Man*p* residues in a species-specific manner. (*b*) Primary structure of an *N*-glycan chain from PDB entry 3d12 (Xu *et al.*, 2008 ▶), in which none of the residues is known at its position in *N*-glycans to date and which probably is based on misinterpretation of the electron density.

**Figure 7:**
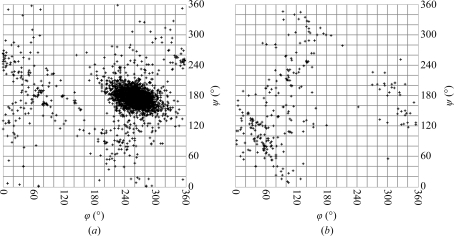
Comparison of observed torsion angles of β-d-Glc*p*NAc-(1–*N*)-Asn (*a*) and α-d-Glc*p*NAc-(1–*N*)-Asn (*b*) linkages. The latter linkage is not known to occur in nature, so that its presence in the PDB is probably based on erroneous coordinates. This assumption is supported by the relatively large scatter of the α-linkages in comparison to the β-linkages. The occurrence of these structures in the PDB might be based on improper or lacking chirality restraints on the linking C atom. The plots were generated with *glyTorsion* (see the legend to Fig. 2 ▶).

Unfortunately, the carbohydrate moieties in the PDB entries contain a rather large number of errors. Some years ago, a systematic study of all carbohydrate-containing PDB entries revealed that about 30% of them contain at least one error such as mismatches between the PDB residue names and the residue actually present in the three-dimensional structure, missing or surplus connectivities or surplus atoms (Lütteke *et al.*, 2004 ▶). Not included in that study were *N*-glycan structures, for which there is no biosynthetic pathway known, such as α-­d-Glc*p*NAc instead of β-d-Glc*p*NAc, or even more different residues within the *N*-glycan core (Fig. 6 ▶). Such three-dimensional structures, as well as those comprising monosaccharide units with very unusual and probably erroneous ring conformations, provide an additional number of errors in the carbohydrate structures in the PDB (Petrescu *et al.*, 1999 ▶; Crispin *et al.*, 2007 ▶; Nakahara *et al.*, 2008 ▶). Of course, entries containing *N*-glycan chains for which there is as yet no biosynthetic pathway known could indicate new so far undiscovered pathways. Recently, for example, α-d-Gal*p*NAc and β-d-6-deoxy-Glc*p*NAc4NAc (‘bacillosamine’; β-d-Bac*p*) were found in a bacterial *N*-glycan core (Young *et al.*, 2002 ▶). However, when comparing the ϕ/ψ plots of the glycosidic torsions of β-d-Glc*p*NAc-(1–*N*)-Asn and α-d-Glc*p*NAc-(1–*N*)-Asn linkages it becomes obvious that the torsions of the latter type of linkage are significantly more widely scattered (Fig. 7 ▶). This is what one would expect for erroneous linkages, indicating that they are indeed most likely to be incorrect three-dimensional structures. This kind of error might be caused by improper or lacking chirality constraints on the linking C atom or by electron density being modelled without enough regard to known chemistry (Crispin *et al.*, 2007 ▶; Berman *et al.*, 2007 ▶).

**Figure 8:**
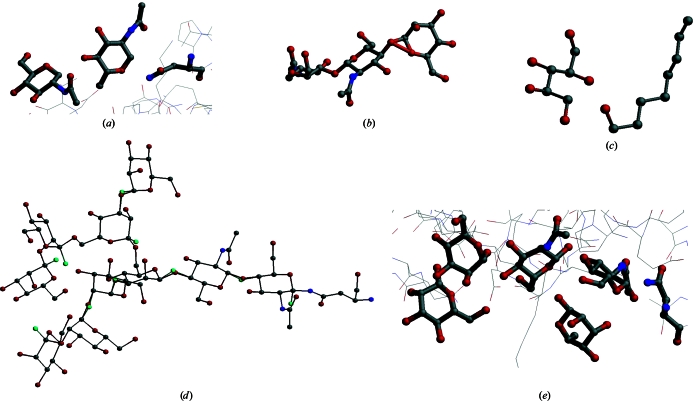
Examples of errors in carbohydrate chains in the PDB. (*a*) Missing LINK records can result in too large distances between individual residues (PDB entry 1eqh; Selinsky *et al.*, 2001 ▶). (*b*) Superfluous LINK records can be found when nonlinked atoms are rather close in space (PDB entry 1apy; Oinonen *et al.*, 1995 ▶). (*c*) In entry 1pxx (Rowlinson *et al.*, 2003 ▶), the C_1_ atom is missing, although all three surrounding atoms are resolved in the three-dimensional structure. (*d*) Superfluous atoms (cyan) are sometimes found within glycosidic linkages (Dellisanti *et al.*, 2007 ▶). (*e*) When individual unconnected residues are arranged in a way that is usually found in *N*-glycan chains, they probably should be linked to each other, which would result in a deletion of the O_1_ atoms (PDB entry 3d2u; Yang & Bjorkman, 2008 ▶).

Another frequent type of errors within the carbohydrate parts of PDB entries is related to the connections between atoms or residues. Superfluous entries in the CONECT records of a PDB file can lead to rather weird-looking structures and missing CONECT records can also cause problems for programs that rely on these records. Many programs, however, assign the connections between atoms by a distance-based approach or use residue libraries to assign connections of atoms within individual residues. Connections between separate residues, however, cannot be covered by residue libraries. Therefore, the correctness and completeness of the LINK records, which contain the information on inter-residue linkages (*i.e.* glycosidic linkages for carbohydrates), is much more essential than that of the CONECT records. Missing linkage information, for example, can induce refinement programs to pull residues apart. This will result in monosaccharide units with anomeric centres that are lacking a bond to an exocyclic O atom or a respective atom and thus seem to be ‘1-deoxy’ residues (Fig. 8 ▶ *a*). Superfluous LINK records are mainly found in structures which contain nonlinked atoms at rather close distances to each other (Fig. 8 ▶ *b*). In contrast to missing LINK records, missing atoms cannot generally be considered as an error, as residues might be only partially resolved in electron-density maps. In some entries, however, there are atoms missing with all the surrounding atoms present in the PDB file (Fig. 8 ▶ *c*). In such cases, the missing atoms should be considered as an error. In some glycosidic linkages, superfluous atoms are found. Linking a monosaccharide to an amino acid or another carbohydrate residue is a condensation reaction, *i.e.* the anomeric O atom is released as a water molecule and the anomeric C atom is linked to an O, N or S atom of the amino acid or the other carbohydrate residue. In some PDB entries, however, the anomeric O atoms are still present within some linkages, sometimes overlapping with the respective atom of the previous residue and sometimes in the position of the H atom that is connected to the anomeric C atom (Fig. 8 ▶ *d*). When such superfluous atoms and missing LINK records occur together on the same residue, the problem is difficult to detect: in some PDB entries, there are individually complete monosaccharides present which are not linked to the protein or to each other, but the anomeric centre of one of the d-Glc*p*NAc residues is in close proximity to the N^δ2^ atom of an Asn side chain which is part of an Asn-Xaa-Ser/Thr sequon and the individual monosaccharide units are arranged in the way in which they are usually present in *N*-­glycan chains (Fig. 8 ▶ *e*). In such cases, it is very likely that they are actually meant to be linked to each other or the protein, which is sometimes confirmed by the respective publication, which mentions *N*-­glycosylation of the protein (Yang & Bjorkman, 2008 ▶).

A frequent issue with carbohydrate residues in PDB entries is mismatches between the PDB residue name and the residue type present in the coordinates. The most common problem of this type is the use of the residue name MAN, which is defined in PDB files as α-d-Man*p*, for β-d-Man*p* residues. However, the latter residues should be named BMA according to the PDB residue definitions. There are 705 nonremediated PDB entries that contain a total of 1585 β-d-Man*p* residues. Of these, 1206 residues in 542 entries are wrongly named MAN, while only 379 residues in 167 entries are correctly called BMA. In contrast, there are only 25 α-d-Man*p* residues in 14 PDB entries that are wrongly named BMA, while 2555 residues of this type in 817 entries are correctly assigned as MAN. Most of these mismatches were corrected during the remediation of the PDB (Henrick *et al.*, 2008 ▶), but this kind of mismatch still frequently occurs in PDB entries that have been published after the remediation date. One reason for the high frequency of mismatched residue names might be the fact that the PDB file format allows only three characters for residue names, which is sufficient for amino acids or nucleotides but results in rather cryptic names for most carbohydrate residues. Monosaccharide notation usually results in longer residue names (for more information on carbohydrate notation, see McNaught, 1997 ▶). Furthermore, there used to be many ambiguities and redundancies within the PDB residue-name definitions; on one hand many residue names were used, for example, for both the α and the β anomeric form of a monosaccharide, while on the other hand more than one residue name existed for some monosaccharides (Lütteke & von der Lieth, 2004 ▶). These problems have been solved by the redefinition of residue names or by marking some residue names as obsolete, respectively, during the recent remediation of the PDB (Henrick *et al.*, 2008 ▶). However, this does not solve the problem of the rather cryptic three-letter codes used for carbohydrates in PDB files. Therefore, many of the mis­matches between residue names and the residues present in the three-dimensional structural data are probably caused by the selection of the wrong residue name. The name MAN (α-­d-Man*p*), for instance, is rather suggestive of mannose residues, while BMA (β-d-Man*p*) is less easily associated with a mannose. This, together with the fact that there are significantly more cases where MAN is used for β-d-Man*p* than cases where α-d-Man*p* residues are called BMA (see above), suggests that the majority of the former cases are a consequence of wrong notation rather than erroneous coordinates. However, these do exist as well, as indicated by the frequent occurrence of incorrect residues within the *N*-glycan cores (see above). The well defined primary structures of *N*-glycan cores enable a rather easy distinction between wrong names and three-dimensional structure errors within this part of carbohydrate chains. For *O*-glycans, this is often more difficult, as various different types of *O*-glycosylation exist (Spiro, 2002 ▶). Noncovalently bound ligands are even more difficult, as theoretically any residue could be present and thus the decision whether a mismatch is caused by a wrong residue name or erroneous coordinates cannot be made without further knowledge of the experimental conditions (in particular the ligand that was actually used in the experiment).

## 4. Validation tools

**Figure 9:**
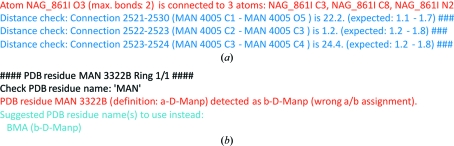
Examples of *pdb-care* error messages. (*a*) Connectivities check. Atoms that are linked to too many other atoms are labelled in red, while bond lengths that are not within a user-defined tolerance range are marked in blue. (*b*) Validation of residue names. Mismatches between the detected residue type and the PDB residue name are listed together with the correct name for the detected residue, if present.

The rather large number of errors in the carbohydrate moieties of PDB entries is caused on one hand by the com­plexity of carbohydrates and on the other by the facts that few validation programs exist and that these are not used by many experimentalists. For the protein parts, various validation tools are well established, such as *WHAT_CHECK* (Hooft *et al.*, 1996 ▶) and *PROCHECK* (Laskowski *et al.*, 1993 ▶). Much later, the first validation programs to be focused on carbohydrates were published. The ***PDBCa**rbohydrate **Re**sidue Check* (*pdb-care*) software (http://www.glycosciences.de/tools/pdb-care/; Lütteke & von der Lieth, 2004 ▶) can perform some checks on connectivities (Fig. 9 ▶ *a*), but the main focus of this tool is to locate mismatches between the carbohydrate residue names that are used in a PDB file and the residue that is actually present in the three-dimensional structure. If mismatches are found, the carbohydrate residue type as detected from the coordinates, the one that is defined by the PDB residue name used and, if present, a PDB residue name that matches the detected residue are displayed to the user (Fig. 9 ▶ *b*). These data help the user to decide whether the residue name has to be changed or whether an error in the coordinates is present. Currently, *pdb-care* does not yet test whether a detected *N*-­glycan structure biologically makes sense, *i.e.* whether there is a biochemical pathway known to synthesize the primary structure of that glycan. Such checks can be performed with the *getCarbo* software (http://www.glycostructures.jp/; Nakahara *et al.*, 2008 ▶), which tries to match the *N*-glycan primary structures present in a PDB file with those stored in the KEGG glycan database (http://www.genome.ac.jp/kegg/glycan/; Hashimoto *et al.*, 2006 ▶) and indicates prob­lems graphically in the results files, which are sent to the user by e-mail.

The torsion angles that determine the conformation of a carbohydrate chain can be evaluated in a way similar to the Ramachandran plot (Ramachandran *et al.*, 1963 ▶), which is a frequently used method to evaluate the quality of the protein backbone conformation (Hooft *et al.*, 1997 ▶; Lovell *et al.*, 2003 ▶). As described in §[Sec sec2.2]2.2, the preferred conformations of a glycosidic linkage depend on the residues involved and the linkage type. Therefore, in contrast to the protein Ramachandran plot, one cannot plot all torsion angles observed in one three-dimensional structure onto one single map. Instead, various residue- and position-dependent plots are needed. These are generated by the *carp* (*Carbohydrate Ramachandran Plot*) software (www.glycosciences.de/tools/carp/; Lütteke *et al.*, 2005 ▶). To judge the quality of the observed torsions, comparison data are needed. These can be retrieved from the carbohydrate torsions that are present in the PDB as provided by *glyTorsion* (http://www.glycosciences.de/tools/glytorsion/) or from computationally generated maps retrieved from the GlycoMapsDB (http://www.glycosciences.de/modeling/glycomapsdb/; Frank *et al.*, 2007 ▶). As carbohydrate chains are rather flexible, linkages that are not present in the preferred conformation are not necessarily erroneous. Interactions with the protein surface, such as hydrogen bonds, stacking interactions or sterical hindrance, can promote a conformation that is less favourable in solution or in other glycoproteins or protein–carbohydrate complexes. Nevertheless, the carbo­hydrate Ramachandran plot can be a useful tool to identify unusual and thus potentially erroneous conformations.

In addition to the software that has primarily been written for the validation of carbohydrate three-dimensional structures, there are a number of further tools and databases available that are focused on carbohydrates and can support researchers who are working with carbohydrate three-dimensional structures. The glycosciences.de database (http://www.glycosciences.de/sweetdb/; Lütteke *et al.*, 2006 ▶) and the GDB:Structures database (http://www.glycostructures.jp; Nakahara *et al.*, 2008 ▶) can be searched for PDB entries that contain specific carbohydrate chains; KEGG Pathway (http://www.genome.jp/kegg/pathway.html#glycan; Kanehisa *et al.*, 2006 ▶) and the glycosyltransferase database of the Con­sortium for Functional Glycomics (http://www.functionalglycomics.org/glycomics/molecule/jsp/glycoEnzyme/geMolecule.jsp; Raman *et al.*, 2005 ▶) provide information on known biosynthetic pathways for glycan biosynthesis. A more thorough overview of freely available web resources related to glycobiology has recently been published elsewhere (Lütteke, 2008 ▶).

## 5. Conclusions

With more than 3500 entries for glycoproteins or protein–carbohydrate complexes, the PDB forms a valuable resource for glycoscientists. Insights into the molecular basis of how glycosylation influences protein properties as well as into specific interactions between proteins and carbohydrate ligands can be gained from the three-dimensional structural data. Furthermore, these data provide information on the general properties of carbohydrate chains, such as preferred conformations. Unfortunately, many errors and problems occur within the carbohydrate moieties of these PDB entries. Many of these issues can be detected automatically with the recently developed validation tools, so that researchers that do not have much experience with glycobiology can also easily locate problems within the carbohydrate moieties of three-dimensional structures. This can help users of the PDB to find high-quality structures, *e.g.* for further use in MD simulations, but in particular can help the depositors of three-dimensional structures to detect errors before they submit their coordinates to the PDB. Therefore, the frequent use of carbo­hydrate-validation tools can help to increase the quality of the carbohydrate three-dimensional structures that are present in the PDB.

## Figures and Tables

**Table 1:** Overview of frequently used definitions of glycosidic torsion angles

Angle	NMR style	C − 1 crystallographic style	C + 1 crystallographic style
ϕ	H_1_—C_1_—O—C′_*x*_	O_5_—C_1_—O—C′_*x*_	O_5_—C_1_—O—C′_*x*_
ψ	C_1_—O—C′_*x*_—H′_*x*_	C_1_—O—C′_*x*_—C′_*x*−1_	C_1_—O—C′_*x*_—C′_*x*+1_
ψ [(1–6)-linkage]	C_1_—O—C′_6_—C′_5_	C_1_—O—C′_6_—C′_5_	C_1_—O—C′_6_—C′_5_
ω	O—C′_6_—C′_5_—H′_5_	O—C′_6_—C′_5_—C′_4_	O—C′_6_—C′_5_—O′_5_

**Table 2:** Overview of the numbers of carbo­hydrate-containing entries, carbo­hydrate chains and residues (monosaccharide units) found in the PDB (adopted from Lütteke & Frank, 2009 ▶) The count values are based on the PDB release of March 2008, which contained about 50 000 entries.

	*N*-glycan	*O*-glycan	Ligand	Total
Entries	1595	182	2142	3561
Chains	6398	783	5277	12458
Residues	12399	912	9400	22711

**Table 3:** Chain length (number of residues in the chain) of carbohydrate chains in the PDB The table lists the number of chains of a certain length found in the PDB release of March 2008 (adopted from Lütteke & Frank, 2009 ▶).

Length	*N*-glycan	*O*-glycan	Ligand	Total
1	3575	707	3044	7326
2	1456	50	1240	2746
3	672	13	504	1189
4	203	5	205	413
5	184	4	176	364
6	143	2	53	198
7	74	2	24	100
8	49	—	17	66
9	35	—	5	40
10	6	—	3	9
11	1	—	3	4
12	—	—	1	1
15	—	—	2	2

## References

[bb1] Ali, M. M., Aich, U., Varghese, B., Perez, S., Imberty, A. & Loganathan, D. (2008). *J. Am. Chem. Soc.* **130**, 8317–8325. 10.1021/ja800335m 18540582

[bb2] Allen, F. H. (2002). *Acta Cryst.* B**58**, 380–388. 10.1107/s0108768102003890 12037359

[bb3] Apweiler, R., Hermjakob, H. & Sharon, N. (1999). *Biochim. Biophys. Acta*, **1473**, 4–8. 10.1016/s0304-4165(99)00165-8 10580125

[bb4] Ashwell, G. & Harford, J. (1982). *Annu. Rev. Biochem.* **51**, 531–554. 10.1146/annurev.bi.51.070182.002531 6287920

[bb5] Bailey, D., Renouf, D. V., Large, D. G., Warren, C. D. & Hounsell, E. F. (2000). *Carbohydr. Res.* **324**, 242–254. 10.1016/s0008-6215(99)00247-5 10744333

[bb6] Beis, K., Srikannathasan, V., Liu, H., Fullerton, S. W., Bamford, V. A., Sanders, D. A., Whitfield, C., McNeil, M. R. & Naismith, J. H. (2005). *J. Mol. Biol.* **348**, 971–982. 10.1016/j.jmb.2005.02.057 PMC3326527 15843027

[bb7] Berman, H. M., Henrick, K., Nakamura, H. & Markley, J. (2007). *Nature Struct. Mol. Biol.* **14**, 354–355.

[bb8] Berman, H. M., Westbrook, J., Feng, Z., Gilliland, G., Bhat, T. N., Weissig, H., Shindyalov, I. N. & Bourne, P. E. (2000). *Nucleic Acids Res.* **28**, 235–242. 10.1093/nar/28.1.235 PMC102472 10592235

[bb9] Berteau, O. & Stenutz, R. (2004). *Carbohydr. Res.* **339**, 929–936. 10.1016/j.carres.2003.11.008 15010300

[bb10] Bohne-Lang, A., Lang, E., Forster, T. & von der Lieth, C. W. (2001). *Carbohydr. Res.* **336**, 1–11. 10.1016/s0008-6215(01)00230-0 11675023

[bb11] Bosques, C. J., Tschampel, S. M., Woods, R. J. & Imperiali, B. (2004). *J. Am. Chem. Soc.* **126**, 8421–8425. 10.1021/ja049266 PMC1386730 15237998

[bb12] Brinkman-van der Linden, E. C., de Haan, P. F., Havenaar, E. C. & van Dijk, W. (1998). *Glycoconj. J.* **15**, 177–182. 10.1023/a:1006972307166 9557878

[bb13] Brisson, J. R. & Carver, J. P. (1983). *Biochemistry*, **22**, 3671–3680. 10.1021/bi00284a021 6615790

[bb14] Casasnovas, J. M., Springer, T. A., Liu, J. H., Harrison, S. C. & Wang, J. H. (1997). *Nature (London)*, **387**, 312–315. 10.1038/387312a0 9153399

[bb15] Chang, V. T., Crispin, M., Aricescu, A. R., Harvey, D. J., Nettleship, J. E., Fennelly, J. A., Yu, C., Boles, K. S., Evans, E. J., Stuart, D. I., Dwek, R. A., Jones, E. Y., Owens, R. J. & Davis, S. J. (2007). *Structure*, **15**, 267–273. 10.1016/j.str.2007.01.011 PMC1885966 17355862

[bb16] Charlwood, J., Bryant, D., Skehel, J. M. & Camilleri, P. (2001). *Biomol. Eng.* **18**, 229–240. 10.1016/s1389-0344(01)00098-3 11911090

[bb17] Chatwell, L., Holla, A., Kaufer, B. B. & Skerra, A. (2008). *Mol. Immunol.* **45**, 1981–1994. 10.1016/j.molimm.2007.10.030 18061677

[bb18] Crispin, M., Stuart, D. I. & Jones, E. Y. (2007). *Nature Struct. Mol. Biol.* **14**, 354. 10.1038/nsmb0507-354a 17473875

[bb19] Cumming, D. A. & Carver, J. P. (1987). *Biochemistry*, **26**, 6676–6683. 10.1021/bi00395a017 3427036

[bb20] Cumming, D. A., Shah, R. N., Krepinsky, J. J., Grey, A. A. & Carver, J. P. (1987). *Biochemistry*, **26**, 6655–6663. 10.1021/bi00395a015 3427034

[bb21] Delbaere, L. T. (1974). *Biochem. J.* **143**, 197–205. 10.1042/bj1430197 PMC1168368 4464850

[bb22] Dellisanti, C. D., Yao, Y., Stroud, J. C., Wang, Z. Z. & Chen, L. (2007). *Nature Neurosci.* **10**, 953–962. 10.1038/nn1942 17643119

[bb23] Diekman, A. B. (2003). *Cell. Mol. Life. Sci.* **60**, 298–308. 10.1007/s000180300025 PMC11146057 12678495

[bb24] Drescher, B., Witte, T. & Schmidt, R. E. (2003). *Immunology*, **110**, 335–340. 10.1046/j.1365-2567.2003.01743.x PMC1783064 14632661

[bb25] Duncan, K. (2004). *Curr. Pharm. Des.* **10**, 3185–3194. 10.2174/1381612043383223 15544508

[bb26] Eklund, R., Lycknert, K., Söderman, P. & Widmalm, G. (2005). *J. Phys. Chem. B*, **109**, 19936–19945. 10.1021/jp053198o 16853578

[bb27] Esko, J. D. & Selleck, S. B. (2002). *Annu. Rev. Biochem.* **71**, 435–471. 10.1146/annurev.biochem.71.110601.135458 12045103

[bb28] Frank, M., Bohne-Lang, A., Wetter, T. & Lieth, C. W. (2002). *In Silico Biol.* **2**, 427–439. 12542425

[bb29] Frank, M., Lütteke, T. & von der Lieth, C. W. (2007). *Nucleic Acids Res.* **35**, 287–290. 10.1093/nar/gkl907 PMC1899098 17202175

[bb30] Freeze, H. H. (2006). *Nature Rev. Genet.* **7**, 537–551. 10.1038/nrg1894 16755287

[bb31] Fry, E. E., Newman, J. W., Curry, S., Najjam, S., Jackson, T., Blakemore, W., Lea, S. M., Miller, L., Burman, A., King, A. M. & Stuart, D. I. (2005). *J. Gen. Virol.* **86**, 1909–1920. 10.1099/vir.0.80730-0 15958669

[bb32] Garner, B., Merry, A. H., Royle, L., Harvey, D. J., Rudd, P. M. & Thillet, J. (2001). *J. Biol. Chem.* **276**, 22200–22208. 10.1074/jbc.M102150200 11294842

[bb33] Guo, Y., Feinberg, H., Conroy, E., Mitchell, D. A., Alvarez, R., Blixt, O., Taylor, M. E., Weis, W. I. & Drickamer, K. (2004). *Nature Struct. Mol. Biol.* **11**, 591–598. 10.1038/nsmb784 15195147

[bb34] Hakomori, S. (2002). *Proc. Natl Acad. Sci. USA*, **99**, 10231–10233. 10.1073/pnas.172380699 PMC124893 12149519

[bb35] Haltiwanger, R. S. & Lowe, J. B. (2004). *Annu. Rev. Biochem.* **73**, 491–537. 10.1146/annurev.biochem.73.011303.074043 15189151

[bb36] Hart, G. W., Housley, M. P. & Slawson, C. (2007). *Nature (London)*, **446**, 1017–1022. 10.1038/nature05815 17460662

[bb37] Hashimoto, K., Goto, S., Kawano, S., Aoki-Kinoshita, K. F., Ueda, N., Hamajima, M., Kawasaki, T. & Kanehisa, M. (2006). *Glycobiology*, **16**, 63R–70R. 10.1093/glycob/cwj010 16014746

[bb38] Helenius, A. & Aebi, M. (2001). *Science*, **291**, 2364–2369. 10.1126/science.291.5512.2364 11269317

[bb39] Henrick, K. *et al.* (2008). *Nucleic Acids Res.* **36**, D426–D433.

[bb40] Hooft, R. W., Sander, C. & Vriend, G. (1997). *Comput. Appl. Biosci.* **13**, 425–430. 10.1093/bioinformatics/13.4.425 9283757

[bb41] Hooft, R. W., Sander, C., Vriend, G. & Abola, E. E. (1996). *Nature (London)*, **381**, 272. 10.1038/381272a0 8692262

[bb42] Höög, C., Landersjö, C. & Widmalm, G. (2001). *Chemistry*, **7**, 3069–3077. 10.1002/1521-3765(20010716)7:14<3069::aid-chem3069>3.0.co;2-a 11495434

[bb43] Imberty, A. & Pérez, S. (1995). *Protein Eng.* **8**, 699–709. 10.1093/protein/8.7.699 8577698

[bb44] Imperiali, B. & O’Connor, S. E. (1999). *Curr. Opin. Chem. Biol.* **3**, 643–649. 10.1016/s1367-5931(99)00021-6 10600722

[bb45] Indyk, K., Olczak, T., Ciuraszkiewicz, J., Watorek, W. & Olczak, M. (2007). *Acta Biochim. Pol.* **54**, 567–573. 17653303

[bb46] Itzstein, M. von (2008). *Curr. Opin. Struct. Biol.* **18**, 558–566. 10.1016/j.sbi.2008.07.006 18706999

[bb47] Jaeken, J. & Matthijs, G. (2001). *Annu. Rev. Genomics Hum. Genet.* **2**, 129–151. 10.1146/annurev.genom.2.1.129 11701646

[bb48] Jain, S., Drendel, W. B., Chen, Z. W., Mathews, F. S., Sly, W. S. & Grubb, J. H. (1996). *Nature Struct. Biol.* **3**, 375–381. 10.1038/nsb0496-375 8599764

[bb49] Janin, J. & Wodak, S. (1978). *J. Mol. Biol.* **125**, 357–386. 10.1016/0022-2836(78)90408-4 731698

[bb50] Jiménez, D., Roda-Navarro, P., Springer, T. A. & Casasnovas, J. M. (2005). *J. Biol. Chem.* **280**, 5854–5861. 10.1074/jbc.M412104200 15545280

[bb51] Jones, A. J., Papac, D. I., Chin, E. H., Keck, R., Baughman, S. A., Lin, Y. S., Kneer, J. & Battersby, J. E. (2007). *Glycobiology*, **17**, 529–540. 10.1093/glycob/cwm017 17331977

[bb52] Jones, J., Krag, S. S. & Betenbaugh, M. J. (2005). *Biochim. Biophys. Acta*, **1726**, 121–137. 10.1016/j.bbagen.2005.07.003 16126345

[bb53] Julenius, K., Mølgaard, A., Gupta, R. & Brunak, S. (2005). *Glycobiology*, **15**, 153–164. 10.1093/glycob/cwh151 15385431

[bb54] Kanehisa, M., Goto, S., Hattori, M., Aoki-Kinoshita, K. F., Itoh, M., Kawashima, S., Katayama, T., Araki, M. & Hirakawa, M. (2006). *Nucleic Acids Res.* **34**, D354–D357. 10.1093/nar/gkj102 PMC1347464 16381885

[bb55] Klement, M. L., Ojemyr, L., Tagscherer, K. E., Widmalm, G. & Wieslander, A. (2007). *Mol. Microbiol.* **65**, 1444–1457. 10.1111/j.1365-2958.2007.05865.x 17697098

[bb56] Kobata, A. & Amano, J. (2005). *Immunol. Cell Biol.* **83**, 429–439. 10.1111/j.1440-1711.2005.01351.x 16033539

[bb57] Kogelberg, H. & Feizi, T. (2001). *Curr. Opin. Struct. Biol.* **11**, 635–643. 10.1016/s0959-440x(00)00259-1 11785767

[bb58] Koles, K., van Berkel, P. H., Pieper, F. R., Nuijens, J. H., Mannesse, M. L., Vliegenthart, J. F. & Kamerling, J. P. (2004). *Glycobiology*, **14**, 51–64. 10.1093/glycob/cwh010 14514717

[bb59] Kooyk, Y. van & Rabinovich, G. A. (2008). *Nature Immunol.* **9**, 593–601. 10.1038/ni.f.203 18490910

[bb60] Kornfeld, R. & Kornfeld, S. (1985). *Annu. Rev. Biochem.* **54**, 631–646. 10.1146/annurev.bi.54.070185.003215 3896128

[bb61] Kowarik, M., Numao, S., Feldman, M. F., Schulz, B. L., Callewaert, N., Kiermaier, E., Catrein, I. & Aebi, M. (2006). *Science*, **314**, 1148–1150. 10.1126/science.1134351 17110579

[bb62] Kowarik, M., Young, N. M., Numao, S., Schulz, B. L., Hug, I., Callewaert, N., Mills, D. C., Watson, D. C., Hernandez, M., Kelly, J. F., Wacker, M. & Aebi, M. (2006). *EMBO J.* **25**, 1957–1966. 10.1038/sj.emboj.7601087 PMC1456941 16619027

[bb63] Lahm, H., André, S., Hoeflich, A., Kaltner, H., Siebert, H.-C., Sordat, B., von der Lieth, C. W., Wolf, E. & Gabius, H. J. (2004). *Glycoconj. J.* **20**, 227–238. 10.1023/B:GLYC.0000025817.24297.17 15115907

[bb64] Lakshmanan, T., Sriram, D., Priya, K. & Loganathan, D. (2003). *Biochem. Biophys. Res. Commun.* **312**, 405–413. 10.1016/j.bbrc.2003.10.149 14637152

[bb65] Laskowski, R. A., MacArthur, M. W., Moss, D. S. & Thornton, J. M. (1993). *J. Appl. Cryst.* **26**, 283–291.

[bb66] Lau, K. S., Partridge, E. A., Grigorian, A., Silvescu, C. I., Reinhold, V. N., Demetriou, M. & Dennis, J. W. (2007). *Cell*, **129**, 123–134. 10.1016/j.cell.2007.01.049 17418791

[bb67] Lederkremer, R. M. de & Colli, W. (1995). *Glycobiology*, **5**, 547–552. 10.1093/glycob/5.6.547 8563141

[bb68] Lehr, T., Geyer, H., Maass, K., Doenhoff, M. J. & Geyer, R. (2007). *Glycobiology*, **17**, 82–103. 10.1093/glycob/cwl048 16971380

[bb69] Lieth, C. W. von der, Lütteke, T. & Frank, M. (2006). *Biochim. Biophys. Acta*, **1760**, 568–577. 10.1016/j.bbagen.2005.12.004 16459020

[bb70] Lis, H. & Sharon, N. (1998). *Chem. Rev.* **98**, 637–674. 10.1021/cr940413g 11848911

[bb71] Live, D. H., Kumar, R. A., Beebe, X. & Danishefsky, S. J. (1996). *Proc. Natl Acad. Sci. USA*, **93**, 12759–12761. 10.1073/pnas.93.23.12759 PMC23992 8917491

[bb72] Lommerse, J. P., Kroon-Batenburg, L. M., Kamerling, J. P. & Vliegenthart, J. F. G. (1995). *Biochemistry*, **34**, 8196–8206. 10.1021/bi00025a027 7794934

[bb73] Lommerse, J. P., van Rooijen, J. J., Kroon-Batenburg, L. M., Kamerling, J. P. & Vliegenthart, J. F. (2002). *Carbohydr. Res.* **337**, 2279–2299. 10.1016/s0008-6215(02)00212-4 12433493

[bb74] Loss, A., Bunsmann, P., Bohne, A., Schwarzer, E., Lang, E. & von der Lieth, C. W. (2002). *Nucleic Acids Res.* **30**, 405–408. 10.1093/nar/30.1.405 PMC99123 11752350

[bb75] Lovell, S. C., Davis, I. W., Arendall, W. B. III, de Bakker, P. I., Word, J. M., Prisant, M. G., Richardson, J. S. & Richardson, D. C. (2003). *Proteins*, **50**, 437–450. 10.1002/prot.10286 12557186

[bb76] Lovering, A. L., de Castro, L. H., Lim, D. & Strynadka, N. C. (2007). *Science*, **315**, 1402–1405. 10.1126/science.1136611 17347437

[bb77] Lütteke, T. (2008). *Chembiochem*, **9**, 2155–2160. 10.1002/cbic.200800338 18693281

[bb78] Lütteke, T., Bohne-Lang, A., Loss, A., Goetz, T., Frank, M. & von der Lieth, C. W. (2006). *Glycobiology*, **16**, 71R–81R. 10.1093/glycob/cwj049 16239495

[bb79] Lütteke, T. & Frank, M. (2009). In *Bioinformatics for Glycobiology and Glycomics: An Introduction*, edited by C. W. von der Lieth, T. Lütteke & M. Frank. In the press.

[bb80] Lütteke, T., Frank, M. & von der Lieth, C. W. (2004). *Carbohydr. Res.* **339**, 1015–1020. 10.1016/j.carres.2003.09.038 15010309

[bb81] Lütteke, T., Frank, M. & von der Lieth, C. W. (2005). *Nucleic Acids Res.* **33**, D242–D246. 10.1093/nar/gki013 PMC539967 15608187

[bb82] Lütteke, T. & von der Lieth, C. W. (2004). *BMC Bioinformatics*, **5**, 69. 10.1186/1471-2105-5-69 PMC441419 15180909

[bb83] Marshall, R. (1972). *Annu. Rev. Biochem.* **41**, 673–702. 10.1146/annurev.bi.41.070172.003325 4563441

[bb84] Martinez, V. G., Pellizzari, E. H., Díaz, E. S., Cigorraga, S. B., Lustig, L., Denduchis, B., Wolfenstein-Todel, C. & Iglesias, M. M. (2004). *Glycobiology*, **14**, 127–137. 10.1093/glycob/cwh025 14638631

[bb85] McNaught, A. D. (1997). *Carbohydr. Res.* **297**, 1–92. 10.1016/s0008-6215(97)83449-0 9042704

[bb86] Mendelsohn, R., Cheung, P., Berger, L., Partridge, E., Lau, K., Datti, A., Pawling, J. & Dennis, J. W. (2007). *Cancer Res.* **67**, 9771–9780. 10.1158/0008-5472.CAN-06-4580 17942907

[bb87] Mølgaard, A. & Larsen, S. (2002). *Acta Cryst.* D**58**, 111–119. 10.1107/s0907444901018479 11752785

[bb88] Molinari, M. (2007). *Nature Chem. Biol.* **3**, 313–320. 10.1038/nchembio880 17510649

[bb89] Moody, A. M., Chui, D., Reche, P. A., Priatel, J. J., Marth, J. D. & Reinherz, E. L. (2001). *Cell*, **107**, 501–512. 10.1016/s0092-8674(01)00577-3 11719190

[bb90] Nakahara, S. & Raz, A. (2008). *Anticancer Agents Med. Chem.* **8**, 22–36. 10.2174/187152008783330833 PMC3794466 18220503

[bb91] Nakahara, T., Hashimoto, R., Nakagawa, H., Monde, K., Miura, N. & Nishimura, S. (2008). *Nucleic Acids Res.* **36**, D368–D371. 10.1093/nar/gkm833 PMC2238941 17933765

[bb92] Ohtsubo, K. & Marth, J. D. (2006). *Cell*, **126**, 855–867. 10.1016/j.cell.2006.08.019 16959566

[bb93] Oinonen, C., Tikkanen, R., Rouvinen, J. & Peltonen, L. (1995). *Nature Struct. Biol.* **2**, 1101–1108. 10.1038/nsb1295-1102 8846222

[bb94] Palian, M. M., Jacobsen, N. E. & Polt, R. (2001). *J. Pept. Res.* **58**, 180–189. 10.1034/j.1399-3011.2001.00906.x 11532077

[bb95] Parekh, R. B., Tse, A. G., Dwek, R. A., Williams, A. F. & Rademacher, T. W. (1987). *EMBO J.* **6**, 1233–1244. 10.1002/j.1460-2075.1987.tb02359.x PMC553924 2886334

[bb96] Parodi, A. J. (2000). *Biochem J.* **348**, 1–13. PMC1221029 10794707

[bb97] Petersen, B. O., Sara, M., Mader, C., Mayer, H. F., Sleytr, U. B., Pabst, M., Puchberger, M., Krause, E., Hofinger, A., Duus, J. O. & Kosma, P. (2008). *Carbohydr. Res.* **343**, 1346–1358. 10.1016/j.carres.2008.03.029 18420185

[bb98] Petrescu, A. J., Milac, A. L., Petrescu, S. M., Dwek, R. A. & Wormald, M. R. (2004). *Glycobiology*, **14**, 103–114. 10.1093/glycob/cwh008 14514716

[bb99] Petrescu, A. J., Petrescu, S. M., Dwek, R. A. & Wormald, M. R. (1999). *Glycobiology*, **9**, 343–352. 10.1093/glycob/9.4.343 10089208

[bb100] Ramachandran, G. N., Ramakrishnan, C. & Sasisekharan, V. (1963). *J. Mol. Biol.* **7**, 95–99. 10.1016/s0022-2836(63)80023-6 13990617

[bb101] Raman, R., Raguram, S., Venkataraman, G., Paulson, J. C. & Sasisekharan, R. (2005). *Nature Methods*, **2**, 817–824. 10.1038/nmeth807 16278650

[bb102] Raman, R., Venkataraman, G., Ernst, S., Sasisekharan, V. & Sasisekharan, R. (2003). *Proc. Natl Acad. Sci. USA*, **100**, 2357–2362. 10.1073/pnas.0437842100 PMC151345 12604799

[bb103] Recny, M. A., Luther, M. A., Knoppers, M. H., Neidhardt, E. A., Khandekar, S. S., Concino, M. F., Schimke, P. A., Francis, M. A., Moebius, U., Reinhold, B. B., Reinhold, V. N. & Reinherz, E. L. (1992). *J. Biol. Chem.* **267**, 22428–22434. 1385399

[bb104] Rensburg, S. J. van, Berman, P., Potocnik, F., MacGregor, P., Hon, D. & de Villiers, N. (2004). *Metab. Brain. Dis.* **19**, 89–96. 10.1023/b:mebr.0000027420.50736.62 15214509

[bb105] Rosati, F., Capone, A., Giovampaola, C. D., Brettoni, C. & Focarelli, R. (2000). *Int. J. Dev. Biol.* **44**, 609–618. 11061424

[bb106] Rowlinson, S. W., Kiefer, J. R., Prusakiewicz, J. J., Pawlitz, J. L., Kozak, K. R., Kalgutkar, A. S., Stallings, W. C., Kurumbail, R. G. & Marnett, L. J. (2003). *J. Biol. Chem.* **278**, 45763–45769. 10.1074/jbc.M305481200 12925531

[bb107] Rudd, P. M. & Dwek, R. A. (1997). *Crit. Rev. Biochem. Mol. Biol.* **32**, 1–100. 10.3109/10409239709085144 9063619

[bb108] Rudd, P. M., Morgan, B. P., Wormald, M. R., Harvey, D. J., van den Berg, C. W., Davis, S. J., Ferguson, M. A. & Dwek, R. A. (1997). *J. Biol. Chem.* **272,** 7229–7244. 10.1074/jbc.272.11.7229 9054419

[bb109] Sabesan, S., Bock, K. & Paulson, J. C. (1991). *Carbohydr. Res.* **218**, 27–54. 10.1016/0008-6215(91)84084-r 1802388

[bb110] Sanders, D. A., Staines, A. G., McMahon, S. A., McNeil, M. R., Whitfield, C. & Naismith, J. H. (2001). *Nature Struct. Biol.* **8**, 858–863. 10.1038/nsb1001-858 11573090

[bb111] Schachter, H. (2000). *Glycoconj. J.* **17**, 465–483. 10.1023/a:1011010206774 11421343

[bb112] Schrag, J. D., Procopio, D. O., Cygler, M., Thomas, D. Y. & Bergeron, J. J. M. (2003). *Trends Biochem. Sci.* **28**, 49–57. 10.1016/s0968-0004(02)00004-x 12517452

[bb113] Selinsky, B. S., Gupta, K., Sharkey, C. T. & Loll, P. J. (2001). *Biochemistry*, **40**, 5172–5180. 10.1021/bi010045s 11318639

[bb114] Sharon, N. & Ofek, I. (2000). *Glycoconj. J.* **17**, 659–664. 10.1023/a:1011091029973 11421356

[bb115] Sheik, S. S., Ananthalakshmi, P., Bhargavi, G. R. & Sekar, K. (2003). *Nucleic Acids Res.* **31**, 448–451. 10.1093/nar/gkg084 PMC165531 12520049

[bb116] Shental-Bechor, D. & Levy, Y. (2008). *Proc. Natl Acad. Sci. USA*, **105**, 8256–8261. 10.1073/pnas.0801340105 PMC2448824 18550810

[bb117] Shi, X. & Elliott, R. M. (2004). *J. Virol.* **78**, 5414–5422. 10.1128/JVI.78.10.5414-5422.2004 PMC400336 15113920

[bb118] Shimaoka, M., Xiao, T., Liu, J. H., Yang, Y., Dong, Y., Jun, C. D., McCormack, A., Zhang, R., Joachimiak, A., Takagi, J., Wang, J. H. & Springer, T. A. (2003). *Cell*, **112**, 99–111. 10.1016/s0092-8674(02)01257-6 PMC4372089 12526797

[bb119] Siebert, H.-C., Andre, S., Lu, S. Y., Frank, M., Kaltner, H., van Kuik, J. A., Korchagina, E. Y., Bovin, N., Tajkhorshid, E., Kaptein, R., Vliegenthart, J. F. G., von der Lieth, C. W., Jiménez-Barbero, J., Kopitz, J. & Gabius, H. J. (2003). *Biochemistry*, **42**, 14762–14773. 10.1021/bi035477c 14674750

[bb120] Siebert, H.-C., Born, K., André, S., Frank, M., Kaltner, H., von der Lieth, C. W., Heck, A. J., Jiménez-Barbero, J., Kopitz, J. & Gabius, H. J. (2005). *Chemistry*, **12**, 388–402. 10.1002/chem.200500505 16267866

[bb121] Smith, A. E. & Helenius, A. (2004). *Science*, **304**, 237–242. 10.1126/science.1094823 15073366

[bb122] Smith, B. J., Huyton, T., Joosten, R. P., McKimm-Breschkin, J. L., Zhang, J.-G., Luo, C. S., Lou, M.-Z., Labrou, N. E. & Garrett, T. P. J. (2006). *Acta Cryst.* D**62**, 947–952. 10.1107/S0907444906020063 16929094

[bb123] Spiriti, J., Bogani, F., van der Vaart, A. & Ghirlanda, G. (2008). *Biophys. Chem.* **134**, 157–167. 10.1016/j.bpc.2008.02.005 18329161

[bb124] Spiro, R. G. (2002). *Glycobiology*, **12**, 43R–56R. 10.1093/glycob/12.4.43r 12042244

[bb125] Stevens, J., Corper, A. L., Basler, C. F., Taubenberger, J. K., Palese, P. & Wilson, I. A. (2004). *Science*, **303**, 1866–1870. 10.1126/science.1093373 14764887

[bb126] Suzuki, O. & Abe, M. (2008). *Oncol. Rep.* **19**, 743–748. 18288410

[bb127] Szymanski, C. M. & Wren, B. W. (2005). *Nature Rev. Microbiol.* **3**, 225–237. 10.1038/nrmicro1100 15738950

[bb128] Tenno, M., Ohtsubo, K., Hagen, F. K., Ditto, D., Zarbock, A., Schaerli, P., von Andrian, U. H., Ley, K., Le, D., Tabak, L. A. & Marth, J. D. (2007). *Mol. Cell. Biol.* **27**, 8783–8796. 10.1128/MCB.01204-07 PMC2169402 17923703

[bb129] Tribulatti, M. V., Mucci, J., Cattaneo, V., Agüero, F., Gilmartin, T., Head, S. R. & Campetella, O. (2007). *Glycobiology*, **17**, 1404–1412. 10.1093/glycob/cwm104 17893094

[bb130] Varki, A., Cummings, R., Esko, J., Freeze, H., Hart, G. & Marth, J. (1999). Editors. *Essentials of Glycobiology.* New York: Cold Spring Harbor Laboratory Press. 20301239

[bb131] Viegas, A., Bras, N. F., Cerqueira, N. M., Fernandes, P. A., Prates, J. A., Fontes, C. M., Bruix, M., Romao, M. J., Carvalho, A. L., Ramos, M. J., Macedo, A. L. & Cabrita, E. J. (2008). *FEBS J.* **275**, 2524–2535. 10.1111/j.1742-4658.2008.06401.x 18422658

[bb132] Vijayalekshmi, S., George, S. K., Andersson, L. K., Kihlberg, J. & Baltzer, L. (2003). *Org. Biomol. Chem.* **1**, 2455–2460. 10.1039/b302847j 12956061

[bb133] Vliegenthart, J. F. G. (2006). *FEBS Lett.* **580**, 2945–2950. 10.1016/j.febslet.2006.03.053 16630616

[bb134] Vulliez-Le Normand, B., Saul, F. A., Phalipon, A., Belot, F., Guerreiro, C., Mulard, L. A. & Bentley, G. A. (2008). *Proc. Natl Acad. Sci. USA*, **105**, 9976–9981. 10.1073/pnas.0801711105 PMC2481361 18621718

[bb135] Werz, D. B., Ranzinger, R., Herget, S., Adibekian, A., von der Lieth, C. W. & Seeberger, P. H. (2007). *Am. Chem. Soc. Chem. Biol.* **2**, 685–691. 10.1021/cb700178s 18041818

[bb136] Woods, R. J. (1998). *Glycoconj. J.* **15**, 209–216. 10.1023/a:1006984709892 PMC4201040 9579797

[bb137] Wormald, M. R., Petrescu, A. J., Pao, Y. L., Glithero, A., Elliott, T. & Dwek, R. A. (2002). *Chem. Rev.* **102**, 371–386. 10.1021/cr990368i 11841247

[bb138] Wormald, M. R., Wooten, E. W., Bazzo, R., Edge, C. J., Feinstein, A., Rademacher, T. W. & Dwek, R. A. (1991). *Eur. J. Biochem.* **198**, 131–139. 10.1111/j.1432-1033.1991.tb15995.x 2040275

[bb139] Wyss, D. F., Choi, J. S. & Wagner, G. (1995). *Biochemistry*, **34**, 1622–1634. 10.1021/bi00005a019 7849022

[bb140] Xu, K., Rajashankar, K. R., Chan, Y. P., Himanen, J. P., Broder, C. C. & Nikolov, D. B. (2008). *Proc. Natl Acad. Sci. USA*, **105**, 9953–9958. 10.1073/pnas.0804797105 PMC2474567 18632560

[bb141] Yang, Z. & Bjorkman, P. J. (2008). *Proc. Natl Acad. Sci. USA*, 1**05**, 10095–10100.

[bb142] Ye, Z. & Marth, J. D. (2004). *Glycobiology*, **14**, 547–558. 10.1093/glycob/cwh069 15044398

[bb143] Young, N. M., Brisson, J. R., Kelly, J., Watson, D. C., Tessier, L., Lanthier, P. H., Jarrell, H. C., Cadotte, N., St Michael, F., Aberg, E. & Szymanski, C. M. (2002). *J. Biol. Chem.* **277**, 42530–42539. 10.1074/jbc.M206114200 12186869

[bb144] Yuan, Y., Bleile, D. W., Wen, X., Sanders, D. A., Itoh, K., Liu, H. W. & Pinto, B. M. (2008). *J. Am. Chem. Soc.* **130**, 3157–3168. 10.1021/ja7104152 PMC2788240 18278916

[bb145] Zuylen, C. W. van, Kamerling, J. P. & Vliegenthart, J. F. G. (1997). *Biochem. Biophys. Res. Commun.* **232**, 117–120. 10.1006/bbrc.1997.6241 9125113

